# Relationship between 8-hydroxydeoxyguanosine levels in placental/umbilical cord blood and maternal/neonatal obstetric factors

**DOI:** 10.3892/etm.2012.617

**Published:** 2012-06-20

**Authors:** SATOKO EBINA, TAKAKO CHIBA, TAKASHI OZAKI, IKUO KASHIWAKURA

**Affiliations:** 1 Departments of Disability and Health and; 2 Radiological Life Sciences, Hirosaki University Graduate School of Health Sciences, Hirosaki 036-8564;; 3 Department of Obstetrics and Gynecology, Hirosaki National Hospital, Hirosaki 036-8545, Japan

**Keywords:** placental/umbilical cord blood, oxidative stress, 8-OHdG, smoking, white blood cells

## Abstract

Oxidative stress is associated with the development of various diseases including cancer, arteriosclerosis, diabetes mellitus, hypertension and metabolic syndrome. However, little is known about the involvement of 8-hydroxydeoxyguanosine (8-OHdG) during the perinatal period. At present, few studies have investigated the precise correlations between 8-OHdG levels in cord blood (CB) and the physical conditions of the mother and neonate. To clarify the involvement of 8-OHdG during the perinatal period, the relationships between CB 8-OHdG levels and maternal/neonatal characteristics in vaginal deliveries were determined. The 8-OHdG levels of CB units collected from singleton gestation vaginal deliveries were analyzed. The relationships between 8-OHdG levels and perinatal characteristics were analyzed. The 8-OHdG levels in CB ranged from 0.1 to 1.39 ng/ml (median, 0.37 ng/ml). The relationships between 8-OHdG levels and the perinatal data were analyzed. The 8-OHdG levels detected in the non-smoking group were significantly lower compared to those in the smoking group. However, no significant correlation was observed between 8-OHdG levels and other maternal/ neonatal factors, including umbilical artery acid/base and gas values. Maternal smoking increases the level of the oxidative DNA damage biomarker 8-OHdG in CB. Since oxidative stress may influence the long-term health outcomes of infants after birth, understanding maternal and fetus/neonate stress conditions at delivery may help improve the health of fetuses and infants.

## Introduction

Oxidative stress is defined as an imbalance in the pro-oxidant-antioxidant equilibrium in favor of pro-oxidants (1). It is known that there are relationships between oxidative stress and the development of various diseases such as cancer, arteriosclerosis, diabetes mellitus, hypertension and metabolic syndrome (1–6). The concentration of lipid peroxidation in peripheral blood is generally higher in pregnant women than in non-pregnant women because the rapidly growing fetoplacental unit *in utero* requires a large amount of oxygen (7–9). In addition, pregnancy-induced hypertension is associated with maternal oxidative stress (7,10–12).

Previous studies suggest that an oxidative DNA damage biomarker, 8-hydroxydeoxyguanosine (8-OHdG), is elevated in bladder carcinoma, prostate cancer (13), childhood cancers (14,15), diabetes mellitus (16,17), coronary heart disease (18) and myoma uteri (19), as well as in smokers (20–22). Moreover, the levels of 8-OHdG are higher in males than females (23,24). Maternal mental and physical stress during pregnancy increases not only pregnancy complications, such as miscarriage, premature birth and low birth weight, but also the risk of diseases in later life as well as hematopoietic dysfunction in the fetus/neonate (25,26). Furthermore, physiologically, pregnancy leads to an increase in free radicals due to the high energy demands of maternal physical functions and this causes maternal oxidization damage.

The placental/umbilical cord blood (CB) is the peripheral blood of the fetus; in addition, various hormones and molecules of oxidative stress derived from the maternal body are generally transported into the CB (27). Dziaman *et al* (28) reported that the levels of 8-OHdG in the urine of newborn children are ∼2.5 times higher than those of adult subjects, indicating that 8-OHdG may be a good marker of oxidative stress in newborns. However, little is known about the involvement of 8-OHdG during the perinatal period, and there have been few studies investigating the precise correlations between the levels of 8-OHdG in CB and the physical conditions of the mother and neonate. Knowing the level of 8-OHdG in CB may facilitate long-term health strategies after birth. In the present study, to clarify the involvement of 8-OHdG during the perinatal period, the relationships between the levels of CB 8-OHdG and maternal/neonatal characteristics in vaginal deliveries were determined.

## Materials and methods

### Subjects and cord blood sample collection

Between November 2010 and April 2011, CB units were collected at a single Hospital (Hirosaki National Hospital, Hirosaki, Japan) after obtaining informed consent from all mothers and approval from the Committee of Medical Ethics of Hirosaki National Hospital (Hirosaki, Japan) and the Committee of Medical Ethics of Hirosaki University Graduate School of Medicine (Hirosaki, Japan). The inclusion criteria were singleton gestation vaginal deliveries and birth without resuscitation or immediate rescue procedures. A segment of the umbilical cord was double clamped immediately after neonatal delivery, and the blood was obtained from the umbilical vein before placental delivery (i.e., *in utero* collection). The CB was collected into a sterile collection bag containing 28 ml citrate phosphate dextrose anticoagulant (CBC-20; Nipro, Co., Osaka, Japan) until the flow ceased. A total of 28 CB units were collected and serum was separated within 24 h of CB collection. Eppendorf test tubes filled with separated serum were stored at −80°C until analysis for biochemical parameters. Relevant perinatal data (i.e., maternal age, smoking status, gestational age, duration of labor, birth weight, Apgar score, and umbilical artery acid/base status and gas values) were obtained from hospital records.

### Quantitative analysis of 8-OHdG

The concentration of 8-OHdG in CB was analyzed using highly sensitive 8-OHdG ELISA monitoring kits (Jaica, Fukuroi, Japan). Each assay was performed immediately after thawing of the serum sample. To remove high-molecular-weight proteins, which interfered with the analysis, each CB serum sample was filtered through an ultrafiltration membrane (molecular weight cut-off, 10,000; Amicon). The obtained filtrate was concentrated by a SpeedVac^®^ centrifugal evaporator (Thermo Scientific Savant SPD1010; Thermo Fisher Scientific, Suwanee, GA, USA).

### Statistical analysis

Statistical analysis was performed using SPSS software version 16.0 (SPSS Japan, Inc., Tokyo, Japan) and Origin (OriginLab, Northampton, MA, USA) for Windows. Descriptive statistics are presented as arithmetic median (range). Data were also analyzed by univariate analysis using the Mann-Whitney U test, Kruskal-Wallis test, and Spearman’s rank correlation coefficient depending on the distribution pattern of the data. The level of significance was set at P<0.05.

## Results

### Perinatal data of the study population

The perinatal data of the study population are summarized in Table I. The median maternal age was 30 years; 57.1% of the mothers were non-smokers and 42.9% were smokers. The median gestational duration was 38.5 weeks; 53.6% of the newborn infants were males and 46.4% were females. The median birth weight was 3047 g. The median Apgar scores at 1 and 5 min were both 9. The median umbilical arterial pH was 7.33, and base excess was −2.0 mM/l. The 8-OHdG levels in CB ranged from 0.1 to 1.39 ng/ml (median, 0.37 ng/ml).

### Relationship between 8-OHdG levels and perinatal characteristics

**Figure 1:**
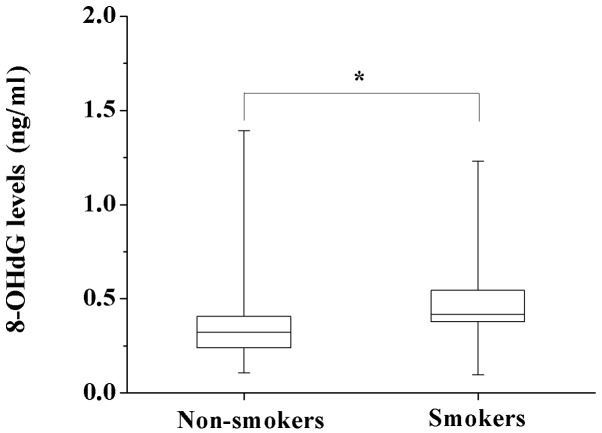
The levels of 8-OHdG detected in the cord blood derived from non-smokers and smokers. The 8-OHdG level detected in the non-smoking group was significantly lower than that in the smoking group (P<0.05).

**Figure 2:**
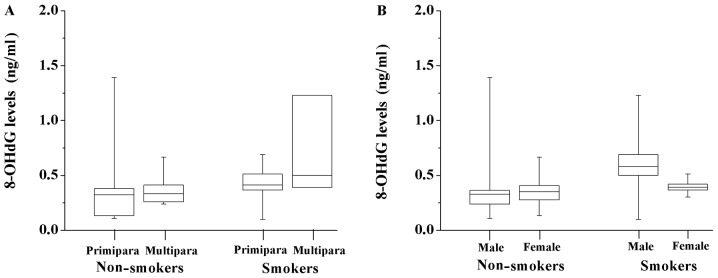
The levels of 8-OHdG detected in the cord blood derived from the primipara and multipara, and male and female subgroups. There were no significant differences between any groups (Kruskal-Wallis test).

The relationships between 8-OHdG levels and perinatal characteristics were analyzed. The 8-OHdG level detected in the non-smoking group was significantly lower than that in the smoking group (0.33 vs. 0.42 ng/ml, P<0.05) (Fig. 1). However, no significant correlation was observed between 8-OHdG levels and other maternal/neonatal factors, including umbilical artery acid/base and gas values. Four cases received oxygen during labor; the 8-OHdG levels in CB ranged from 0.13 to 0.41 ng/ ml. Both above-mentioned groups were further classified as primipara or multipara and male or female. No significant differences were observed between any subgroups (Fig. 2).

The relationships between CB 8-OHdG levels and biochemical markers and blood cells detected in the pre-delivery maternal peripheral blood were also analyzed. A significant positive correlation was found between 8-OHdG level and maternal white blood cells (data not shown). However, no significant correlation was observed between 8-OHdG level and maternal C-reactive protein (CRP), which is an inflammatory marker.

## Discussion

In the present study, the relationships between CB 8-OHdG levels and perinatal maternal/neonatal characteristics were determined. 8-OHdG is formed when DNA is oxidatively modified by reactive oxygen species (ROS). Thus, 8-OHdG is one of the most sensitive biomarkers of oxidative stress and is, therefore, widely used as a biomarker of oxidative DNA damage (29). Forlenza and Miller (30) report that serum 8-OHdG levels are much lower than urine 8-OHdG levels; serum 8-OHdG levels are ∼0.20 to 1.26 ng/ml in healthy adults. In addition, Schulpis *et al* (31) compared the 8-OHdG levels in CB between vaginal and Cesarean section deliveries; the mean 8-OHdG level was 0.25–0.27 ng/ml, indicating that there was no significant difference between delivery modes. In the present study, the 8-OHdG levels in CB obtained from singleton gestation vaginal deliveries ranged from 0.10 to 1.39 ng/ml (Table I), which is the same as that detected in healthy adult serum. In addition, the 8-OHdG levels in smokers were higher than those in non-smokers (Fig. 1). Tobacco causes the creation of ROS, which lead to DNA damage; this subsequently elevates 8-OHdG levels in urine and serum (20–22). Previous studies demonstrate that maternal smoking increases the risk of childhood disorders including preterm delivery (32), intrauterine growth retardation (IUGR), low birth weight (32,33), cleft lip/cleft palate (34), congenital heart disease (35) and attention-deficit hyperactivity disorder (36). Furthermore, some studies report that 8-OHdG levels are elevated in childhood cancer (14), diabetes mellitus (16,17) and coronary heart disease (18). The results of the present study do not indicate any relationships between 8-OHdG levels and various indicators of fetal developmental outcomes. However, additional approaches regarding the characteristics of oxidative stress during the perinatal period are required to improve the health and developmental outcomes of fetuses and infants.

Maternal leukocyte count is well known to increase during pregnancy. Although the mechanism underlying the increase in leukocytes is unclear, it is reported that the count is affected by cortisol, which increases during pregnancy and as a result of various placenta-derived cytokines (37). In the present study, a significant positive correlation was observed between CB 8-OHdG level and pre-delivery maternal white blood cell count (data not shown); meanwhile, CB 8-OHdG level was not related to CRP, an inflammatory response marker (data not shown). Because maternal leukocyte count was measured at different times depending on individuals, its precise changes were unclear. However, there may be a relationship between the number of leukocytes and 8-OHdG level as a result of maternal oxidative stress.

In conclusion, the results of the present study indicate that CB 8-OHdG levels in smokers are significantly higher than those in non-smokers. However, no relationship was found between 8-OHdG level and quantity or duration of smoking. Oxidative stress possibly influences the long-term health outcomes of infants. Although 8-OHdG is a relatively stable chemical substance, it can lead to G→T mutations during DNA replication if generated near chromosomal DNA, increasing the risk of cancer (2). Future investigations focusing on leukocytes, 8-OHdG level, and CB inflammatory cytokine levels are required. Healthcare personnel involved in perinatal medicine should try to reduce oxidative stress during pregnancy to improve the health of the fetus after birth.

## Figures and Tables

**Table I:** Perinatal characteristics of the study population.

Maternal factors	Median (range)

Maternal age (years)	30.0 (16–42)
Gestational age (weeks)	38.5 (32–41)
Maternal smoking status	
Non-smokers, n (%)	16 (57.1)
Smokers, n (%)	12 (42.9)
Parity	
Primipara, n (%)	20 (71.4)
Multipara, n (%)	8 (28.6)
Total duration of labor (min)	360.5 (50–2664)
First stage of labor (min)	279.5 (38–2497)
Second stage of labor (min)	24.0 (1–181)
Oxygen administration, n (%)	4 (14.3)

Neonatal factors	Median (range)

Birth weight (g)	3047.0 (2038–3764)
Placental weight (g)	545.0 (400–800)
Neonatal gender	
Males, n (%)	15 (53.6)
Females, n (%)	13 (46.4)
Apgar score	
1 min	9 (8–9)
5 min	9 (8–10)

Placental/umbilical cord blood	Median (range)

pH^a^	7.33 (7.21–7.39)
pCO_2_ (mmHg)^a^	46.7 (33.3–63.9)
pO_2_ (mmHg)^a^	17.0 (8.0–41.0)
HCO_3_^–^ (mmol/l)^a^	24.3 (18.7–28.1)
Base excess (mmol/l)^a^	−2 (−9 to 1)
8-OHdG (ng/ml)^b^	0.37 (0.10–1.39)

Values represent median (range) unless otherwise specified as [n (%)].

a Umbilical artery;

b umbilical vein.
